# Celecoxib alleviates zinc deficiency-promoted colon tumorigenesis through suppressing inflammation

**DOI:** 10.18632/aging.202642

**Published:** 2021-03-03

**Authors:** Xiaolong Yin, Yuting Zhang, Yingling Wen, Yunyao Yang, Hongping Chen

**Affiliations:** 1 Department of Ophthalmology, The Second Affiliated Hospital of Nanchang University, Nanchang 330006, Jiangxi, P.R. China; 2 Department of Histology and Embryology, Medical College, Nanchang University, Nanchang 330006, Jiangxi, P.R. China; 3 Queen Mary School, Medical College, Nanchang University, Nanchang 330006, Jiangxi, P.R. China

**Keywords:** celecoxib, zinc deficiency, colon tumors, Apc^min/+^ mice, inflammatory mediators

## Abstract

Accumulating evidence has shown that dietary zinc deficiency (ZD) increases the risk of various cancers including esophageal and gastric cancer. However, the role of ZD in colon tumorigenesis is unknown and the related mechanisms need to be investigated. *Apc*^min/+^ mice, widely used to mimic the spontaneous process of human intestinal tumor, were used to construct a ZD mice model in this study. Inflammatory mediators such as COX-2, TNF-α, CCL, CXCL, and IL chemokines families were evaluated using real-time PCR and Enzyme-linked immunosorbent assay (ELISA). Besides, the immunoreactivities of cyclin D1, PCNA, and COX-2 in the colon were detected by immunohistochemistry. We found that zinc deficiency could promote colon tumorigenesis in *Apc*^min/+^ mice. The mechanisms are involved in the upregulation of inflammatory mediators: COX-2, TNF-α, CCL, CXCL, and IL chemokines families. Administration of celecoxib, a selective COX-2 inhibitor, decreased colon tumorigenesis in *Apc*^min/+^ mice via inhibiting the inflammatory mediators. ZD plays an important role in the process of colon cancers of *Apc*^min/+^ mice. Celecoxib attenuates ZD-induced colon tumorigenesis in *Apc*^min/+^ mice by inhibiting the inflammatory mediators. Our novel finding would provide potential prevention of colorectal tumor-induced by ZD.

## INTRODUCTION

Colorectal cancer (CRC) ranks among the top three common cancers in humans with or without a familial background [1]. The morbidity of cancer seems to have boomed both in developed and developing countries [2]. Thus, studying the pathogenesis of colon tumorigenesis has become a top priority. Similarly, establishing effective strategies for preventing colorectal cancer appears to be an urgent requirement. It is well known that the absence of adenomatous polyps colis (*Apc*), an inhibitor of WNT signaling, can lead to primary precursor lesion for colon carcinoma in humans [3]. With a germline nonsense mutation in the tumor suppressor gene of *Apc*, *Apc*^min/+^ mice model is a classical model for studying intestinal tumorigenesis [4]. Although previous studies have shown that spontaneous colorectal tumors in *Apc*^min/+^ mice are very rare, they are still a good mice model for studying molecular mechanisms and chemoprophylaxis.

It is generally agreed that colorectal tumorigenesis requires long-term environmental and dietary risk factors. For example, carcinogens, nutrients, and even intestinal flora are involved in tumorigenesis and tumor progression [5]. Zinc, an essential trace element, is related to a variety of diseases including cancer [6]. It has been revealed that zinc deficiency contributes to many types of cancers [2, 7]. Our previous studies have demonstrated that zinc deficiency could promote the proliferation of esophageal cells and enhance the role of NMBA (N-nitrosomethylbenzylamine) in inducing esophageal cancer [8]. It has also indicated that low intracellular zinc level increases the expression of tumorigenic cytokines [6, 9]. However, the effect of zinc deficiency (ZD) on intestinal tumorigenesis is still unclear.

Inflammation is regarded as one of the risk factors for many types of tumors including CRC [10, 11]. Previous data have displayed that zinc deficiency increases expression levels of many inflammatory factors, such as S100a8 (Calgranulin A, MRP8), S100a9 (Calgranulin B, MRP14) and cyclooxygenase-2 (COX-2) [8, 12, 13], which are widely found in the migration and metastasis development of cancer cell [14]. CXCR2, also known as IL-8 RB, belongs to GPCR(G Protein-Coupled Receptors), which is a large family that contains more than 800 receptors in humans and is related to numerous human diseases, and CXCR2 ligands are important in the pathogenesis of various inflammatory diseases [15]. Whereas, the relationship between zinc deficiency-induced inflammation and colorectal tumorigenesis is not clear.

A large number of studies have suggested that nonsteroidal anti-inflammatory drugs (NSAIDs) have dual effects of anti-inflammatory and cancer prevention [16]. COX-2 inhibitors can prevent tumorigenesis by reducing cyclooxygenase activity, decreasing prostaglandin levels and influencing apoptosis [17, 18]. Celecoxib, a selective COX-2 inhibitor, has a stronger tumor inhibitory effect even at low doses [19]. However, the effect of celecoxib on ZD-promoted tumors remains unknown and needs to be explored.

The purpose of this research is to determine the role of ZD in the promotion of intestinal tumors, the expression profile of inflammatory factors and the potential mechanisms of ZD-facilitated intestinal tumors. Our new findings on the inhibitory effects of celecoxib in ZD-promoted tumors may provide a reference for the prevention and treatment of colon cancer.

## RESULTS

### Effects of ZD on the lifespan of *Apc*^min/+^ mice

**Figure 1:**
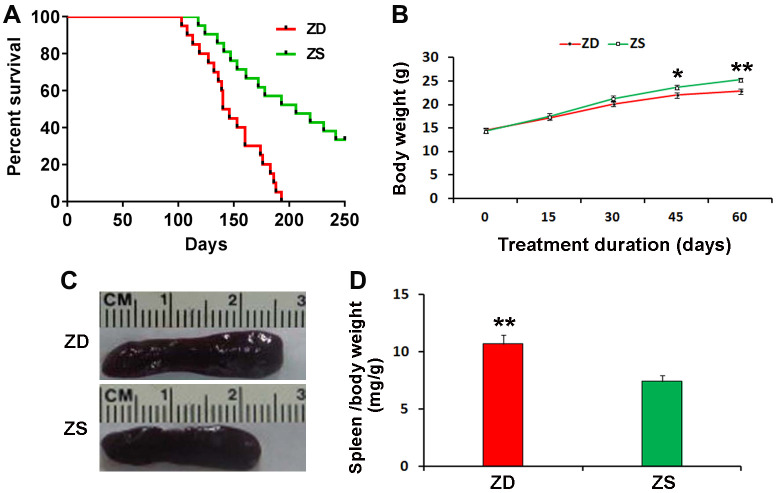
**Effects of zinc deficiency on the lifespan, body weight and spleen index of *Apc^min/+^*.** (**A**) Four weeks old mice were fed with ZD and ZS diets for the long term. Plot evaluated by long-rank test, depicting the percentage survival of mice with ZD (n=20) versus mice with ZS (n=20) surviving longer than the endpoint. (**B**) ZD decreased body weight at the endpoint of ZD or ZS diet fed for the short term. The bodyweight of mice was recorded every 15 days. ZD significantly decreased body weight at diet fed for 45 and 60 days (n=15). (**C**) Representative image of larger spleen ZD group compared with ZS group. (**D**) ZD significantly increased the spleen index. Spleen index (mg/g) was calculated by normalizing spleen weight to body weight at endpoint (n=15). Data were represented as mean ± S.D. *P < 0.05, and **P < 0.01) vs ZS group.

The effect of ZD diet on the lifespan of *Apc*^min/+^ mice was investigated. ZD diet could reduce the survival rate of *Apc*^min/+^ mice. The *Apc*^min/+^ mice of ZD group began to die at 103 days of age and all the mice were dead at 193 days of age. By contrast, the *Apc*^min/+^ mice of ZS diet began to die at 118 days of age, while 50% of mice died at 193 days of age. Thirty percentage of ZS diet *Apc*^min/+^ mice still survived at 250 days of age (Figure 1A).

### Effects of ZD on body weight and spleen index of *Apc*^min/+^ mice

The body weight was monitored. During the 60 days of ZD and ZS diet fed, the body weight was recorded every 15 days. The data indicated that ZD could reduce bodyweight after diet fed for 45 days (Figure 1B).

All mice were sacrificed at the endpoint of the second experiment. The spleen index (spleen weight/ body weight) was calculated. The data showed that ZD significantly increased the weight and index of the spleen. The spleen index of ZD group was 1.44 folds than ZS group (Figure 1C, 1D). It suggests that ZD may be related to the effect of promoting inflammation.

### ZD promoted intestinal tumorigenesis in *Apc*^min/+^ mice

**Figure 2:**
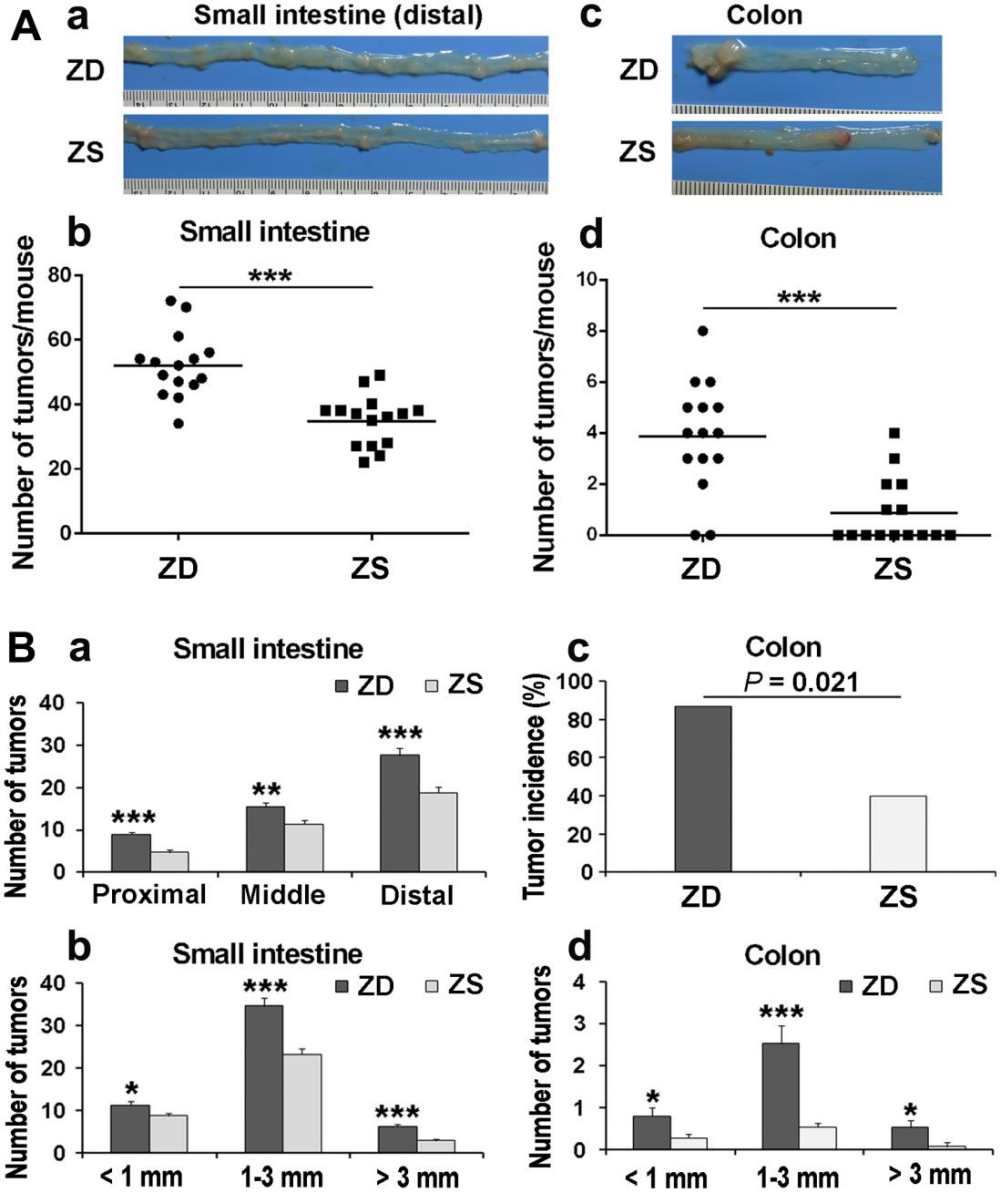
**ZD promoted intestinal tumorigenesis in *Apc^min/+^* mice.** (**A**) The tumor number in the small intestine and colon was calculated after 60 days of ZD or ZS diet fed. (**a**) Representative images of tumor in the distal small intestine. (**b**) Average tumor number is in the small intestine of per mouse. (**c**) Representative image of tumors in the colon. Numbers of tumors from the small intestine per mouse, each point represented one mouse. (**d**) Average tumor number is in the colon per mouse. Horizontal bars indicated the average number. (**B**) Effects of ZD on the tumor multiplicity in small intestine and colon of *Apc*^min/+^. (**a**) Tumor number in proximal, middle, and distal small intestine was evaluated. (**b**) Tumor number in small intestine according to size was analyzed. (**c**) Tumor incidence of the colon in ZD and ZS groups was calculated. (**d**) Tumor number in colon according to size was analyzed. Data are shown as mean ±S.E.M., n=15. *P < 0.05, **P < 0.01 and ***P < 0.001 vs ZS group.

To determine the role of zinc in tumorigenesis, the number and the size of the tumors in the intestine and colon were observed and calculated under the dissecting microscope after 60 days ZD or ZS diet fed. The average number of small intestinal tumors in ZS group was 34.87 per mouse. The average number of tumors was 52.07 per mouse in the ZD group. The number of tumors in ZS group was significantly lower than that in the ZD group (Figure 2A: a, b). Similarly, the average number of tumors in the colon in ZD group (3.87 per mouse) was significantly increased than that in ZS group (0.87 per mouse) (Figure 2A: c, d).

The multiplicity of tumors in the small intestine and colon was also evaluated. According to the length, the small intestine is divided into three segments on average: proximal, middle, and distal segments. In both ZD and ZS groups, the number of tumors in the distal small intestine was the highest. ZD increased the number of tumors in all three segments of the small intestine (Figure 2B: a). The tumor size was classified into three types based on the tumor diameter: small size (< 1 mm), middle size (1-3 mm), and large size (> 3mm). The data indicated that ZD dramatically increased the size of tumors in all three types of tumor size (Figure 2B: b). For tumors in the colon, the tumor incidence in ZD group was also significantly higher than that in ZS group (86.7% vs 40%) (Figure 2B: c). Similarly, ZD also increased the tumor number of all three sizes in the colon (Figure 2B: d). The data above indicated that ZD promoted tumorigenesis in both small intestine and colon.

### ZD induced inflammatory signature

**Figure 3:**
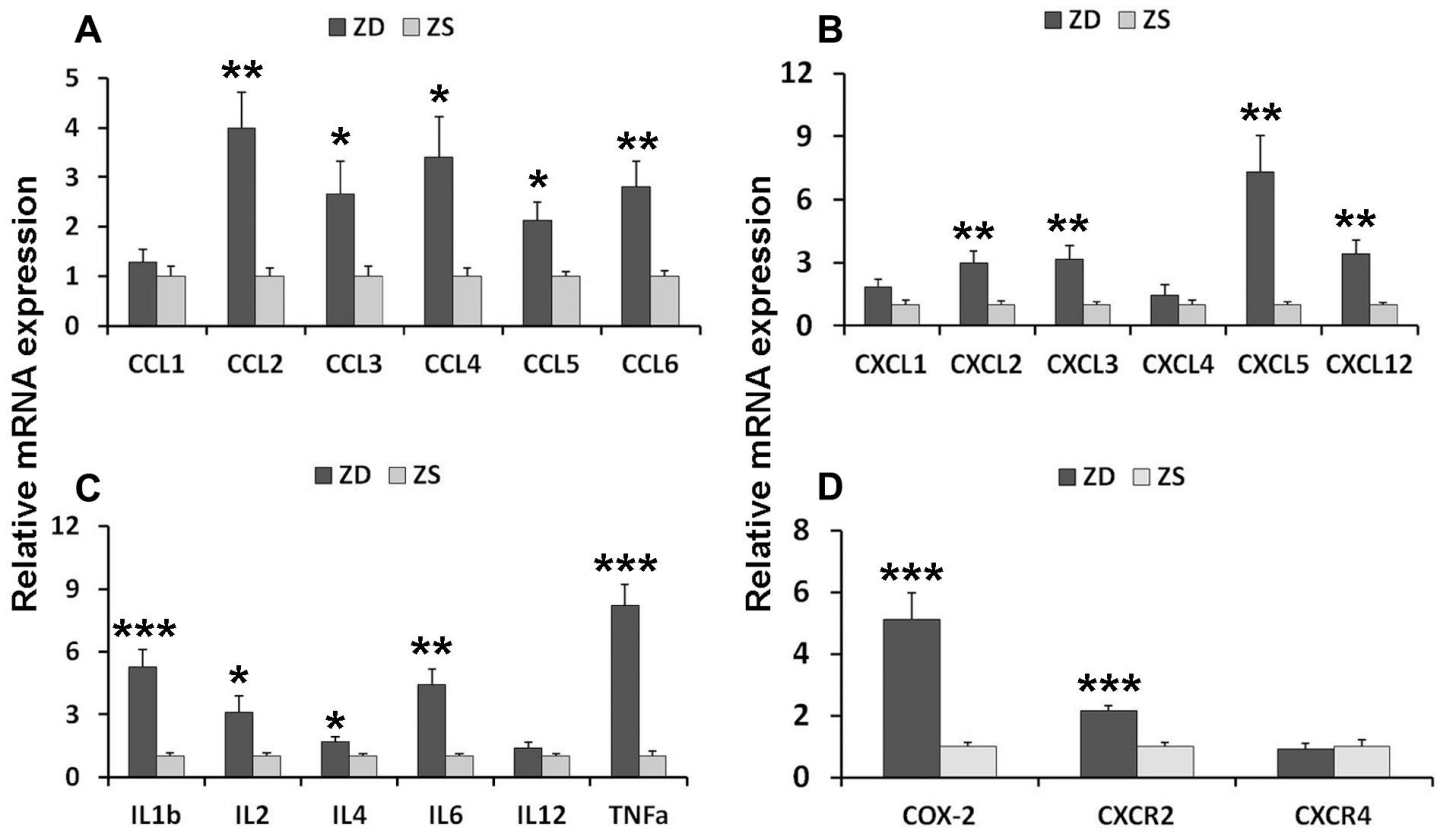
**ZD induced mRNA expression of pro-inflammatory mediators in the colon of *Apc^min/+^* mice.** (**A**) Expression of C-C motif chemokines mRNAs: CCL1, CCL2, CCL3, CCL4, and CCL5. (**B**) Expression of C-X-C motif chemokines mRNAs: CXCL1, CXCL2, CXCL3, CXCL5, and CXCL12. (**C**) Expression of interleukin mRNA: IL-1β, IL-2, IL-4, IL-6 and IL-12, and TNF-α. (**D**) Expression of COX-2, CXCR2, and CXCR4. Data are shown as mean ±S.E.M., n=6. *P < 0.05, **P < 0.01 and ***P < 0.001 vs ZS group.

To investigate the potential mechanism of ZD-induced tumorigenesis, the inflammatory factors and related receptors were analyzed. The expression of levels of mRNA in the intestinal mucosa was detected by real-time PCR. Twenty-one mRNAs were analyzed. The results showed that ZD increased the mRNAs of C-C motif chemokines (CCL2, CCL3, CCL4, and CCL5), C-X-C motif chemokines (CXCL2, CXCL3, CXCL5, and CXCL12), C-X-C motif chemokine receptors CXCR2, Interleukin members (IL-1, IL-2, IL-4, IL-6, and IL-12), TNF-α and COX2 in the colonic mucosa (Figure 3).

**Figure 4:**
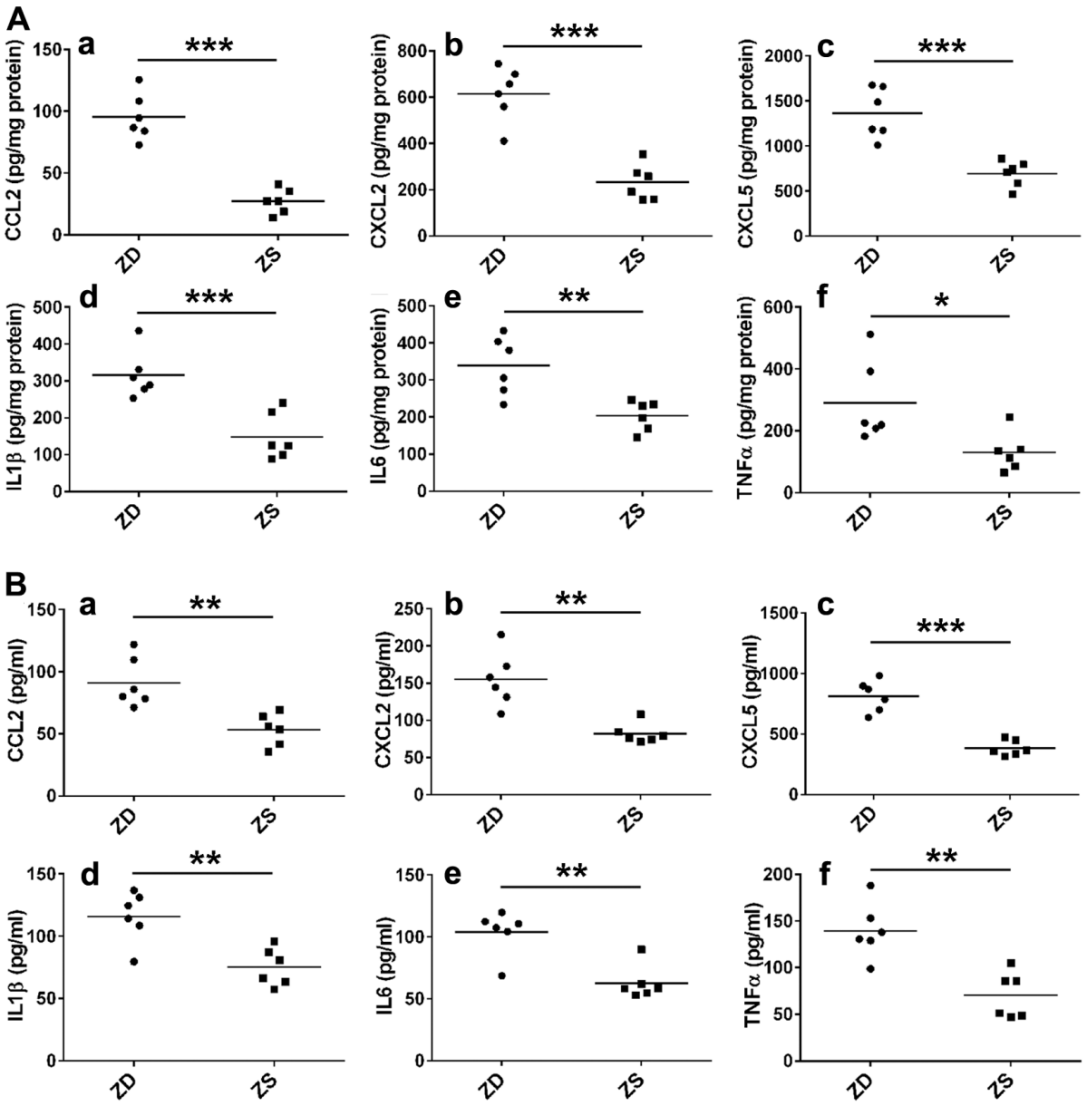
**ZD promoted the inflammation.** (**A**) ZD induced the protein pro-inflammatory mediators in the colon. CCL2, CXCL2, CXCL5, IL-1β, IL6, and TNF-α protein were measured by ELISA. (**B**) ZD induced the protein pro-inflammatory mediators in serum. CCL2, CXCL2, CXCL5, IL-1β, IL6, and TNF-α protein were measured by ELISA. Horizontal bars indicated the average protein levels. *P < 0.05, **P < 0.01, and ***P < 0.001 vs ZS group, n=6.

To further explore the role of inflammatory factors in ZD-promoted intestinal tumors, the protein levels of six selected inflammatory factors in colonic mucosa and serum were measured by ELISA assays. The results indicated that ZD significantly promoted the protein levels of CCL2, CXCL2, CXCL5, IL-1, IL-6, and TNF-α (Figure 4A) in the colonic mucosa. Similarly, ZD increased the protein of these inflammatory factors in the small intestine (Supplementary Figure 1). Additionally, ZD also increased the protein levels of these inflammatory factors in serum (Figure 4B). These data suggested that ZD-fueled inflammation was related to intestinal tumorigenesis.

### ZD increased the immunoreactivities of cyclin D1, PCNA, and COX-2 in the colon of *Apc*^min/+^ mice

**Figure 5:**
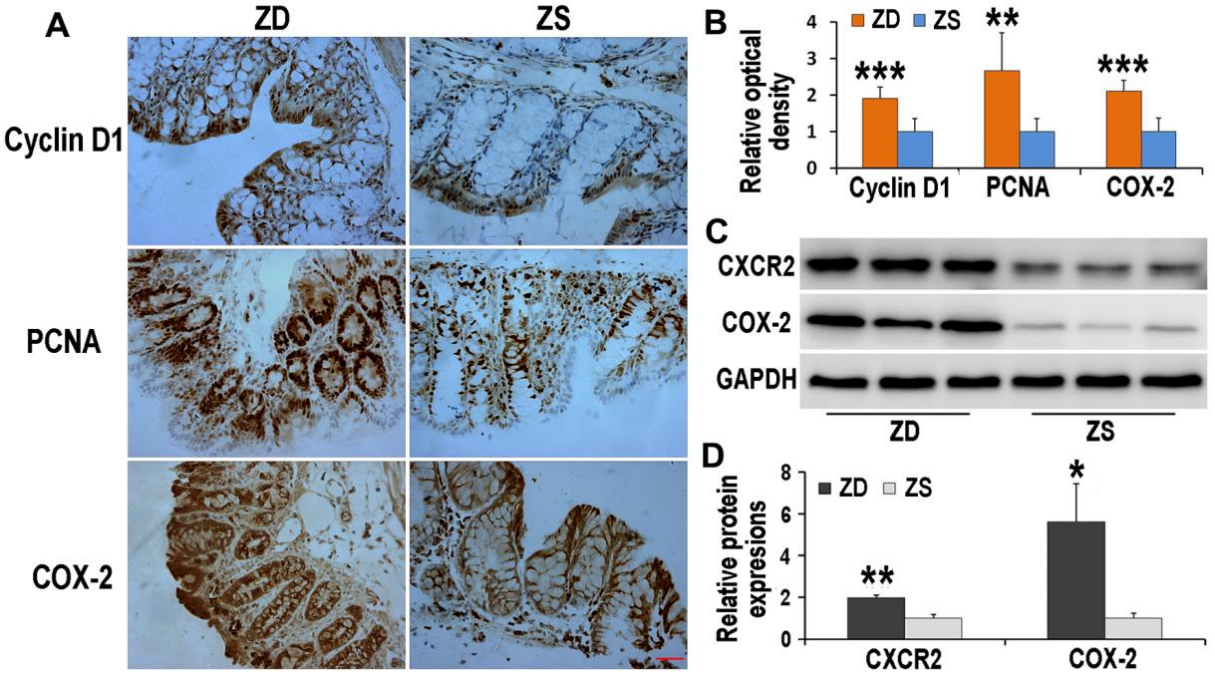
**Effects of ZD on the expressions of Cyclin D1, PCNA, COX-2 and CXCR2.** ZD increased the immunoreactivities of Cyclin D1, PCNA, and COX-2 in the colon of *Apc^min/+^* mice. (**A**) Immunoreactivities of Cyclin D1, PCNA, and COX-2 were detected by immunohistochemistry. (**B**) The data of relative optical density of immunoreactivities. n=6. (**C**) ZD increased expressions of CXCR2 and COX-2 in the colon of *Apc^min/+^* mice. Expressions of CXCR2 and COX-2 were detected by Western blot. GADPH was used to normalize the expression level. (**D**) The data of relative protein expression of Western blot. N=3. Data are showed as mean ± S.D., *P < 0.05, **P < 0.01 and ***P < 0.001 vs ZS groups.

The Immunoreactivities of cyclin D1, PCNA, and COX-2 were detected by immunohistochemistry (Figure 5A). The data indicated that, compared with ZS group, the COX-2 immunoreactivities located on both epithelium and stroma increased in ZD group. Beyond this, both expressions of cyclin D1 and PCNA were up-regulated in ZD diet-fed *Apc*^min/+^ mice. These results implied that ZD had the potential role to promote colon tumors.

### ZD increased the protein levels of CXCR2 and COX-2

The protein expression of CXCR2 and COX-2 was measured by Western blot technique. ZD enhanced the expression levels of CXCR2 and COX-2, both of which are crucial in colon tumorigenesis (Figure 5B).

### Celecoxib attenuated ZD-promoted tumorigenesis by suppressing pro-inflammatory mediators

**Figure 6:**
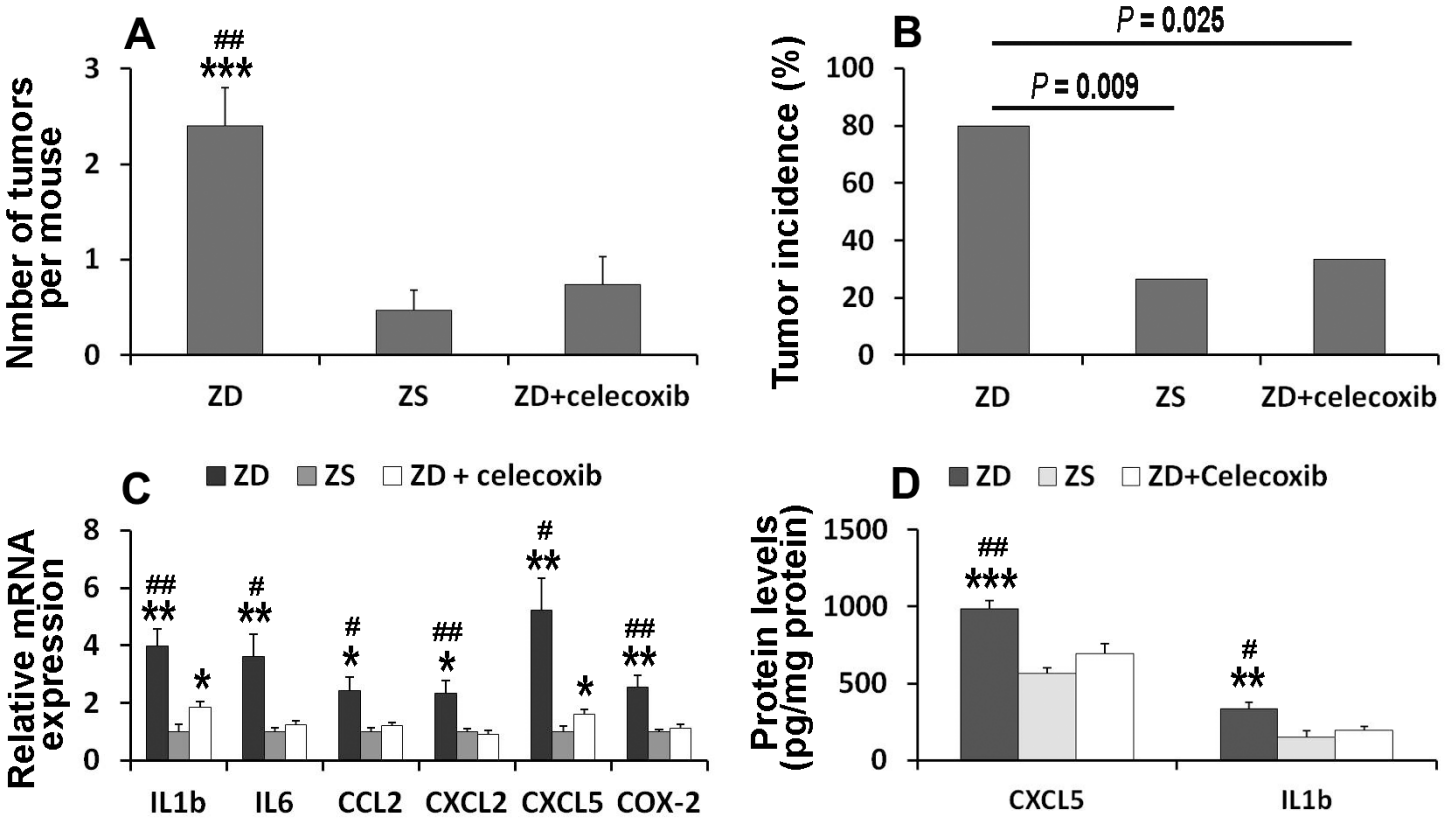
**Celecoxib attenuated ZD-promoted colon tumorigenesis through suppressing pro-inflammatory mediators.** (**A**) Effect of celecoxib on tumor number (n=15). (**B**) Effect of celecoxib on tumor incidence (n=15). (**C**) Effect of celecoxib on mRNA expression of IL1β, IL6, CCL2, CXCL2, and CXCL5 (n=6). (**D**) Effect of celecoxib on protein level of IL1β and CXCL5 (n=6). Data are showed as means ±SEM. *P < 0.05, **P < 0.01 and *** *P* < 0.001 vs ZS groups. ^#^*P* < 0.05 and ^##^*P* < 0.01 vs ZD + celecoxib group.

To clarify whether COX-2 signaling is involved in ZD-induced colon tumorigenesis, celecoxib, a selective COX-2 inhibitor, was orally administrated. The data showed that the number of tumors in ZD, ZS, and ZD + celecoxib groups were 2.4, 0.47, and 0.73, respectively (Figure 6A). The number of tumors per mouse in ZD group was significantly higher than that in ZS and ZD + celecoxib groups. However, there was no significant difference between ZS and ZD + celecoxib groups.

The incidence of tumors in the colon was analyzed. The results showed that the incidence of tumors in ZD, ZS, and ZD + celecoxib groups were 80%, 26.67%, and 33.33%, respectively (Figure 6B). The incidence of tumors was significantly higher in ZD group than that in ZS and ZD + celecoxib groups.

**Figure 7:**
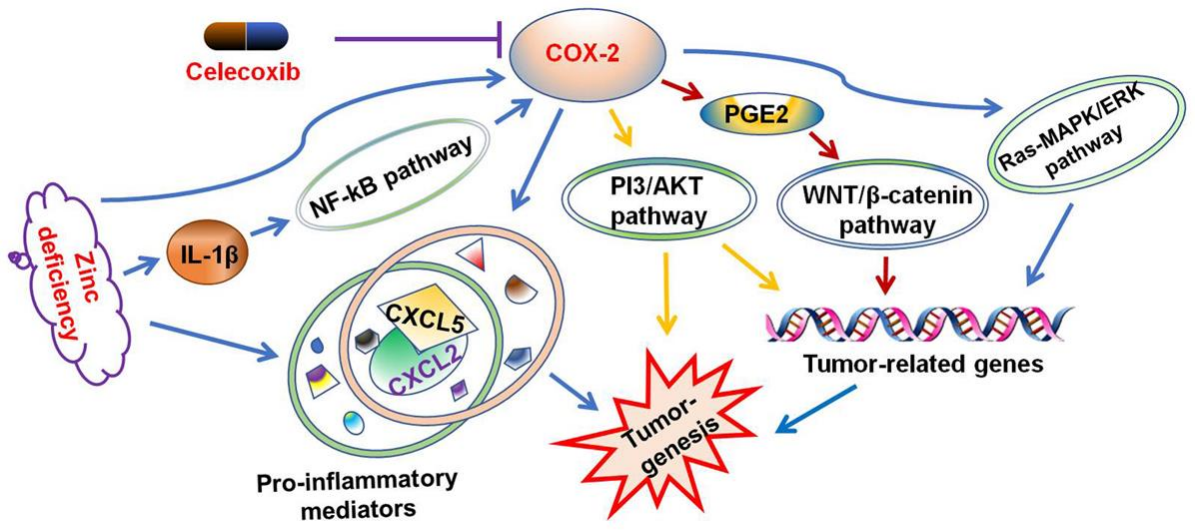
**Celecoxib attenuated ZD-promoted tumorigenesis through suppressing inflammations.** The potential signaling pathway was summarized.

To explore the underlying mechanism of celecoxib in attenuating ZD-promoted tumorigenesis, the expression of mRNA in six pre-inflammatory mediators were evaluated. The data indicated that celecoxib could attenuate the expression of ZD-promoted pre-inflammatory mediators (Figure 6C). In addition, the ELISA assays results showed that celecoxib could decrease the protein concentration of CXCL5 and IL-1 promoted by ZD (Figure 6D). These results confirmed that pre-inflammatory mediators were involved in ZD-promoted colon tumorigenesis. Celecoxib attenuated ZD-promoted tumorigenesis by suppressing inflammations. The potential signaling pathway was shown in Figure 7.

## DISCUSSION

Colorectal cancer is a malignant tumor with increasing morbidity. It can be affected by many factors, such as smoking, drinking, microbial infections, and carcinogens [20, 21]. Nutrition is also involved in promoting the occurrence and development of tumors [22]. There is a link between cancer occurrence and inflammatory factors induced by zinc deficiency [8]. Many researchers have focused on the ZD-related tumorigenesis on esophageal and gastric cancers [23, 24]. However, its influences on colon tumorigenesis and its related mechanism remain unknown.

*Apc*^min/+^ mice are widely used to investigate intestinal tumors. One hundred percent of *Apc*^min/+^ mice can spontaneously develop small intestinal tumors [25]. However, less than 40% of *Apc*^min/+^ mice can develop colorectal tumors. In this study, the role of ZD in *Apc*^min/+^ mice was investigated. The results showed that ZD could shorten the lifespan of mice, reduce their weight, and increase the number and size of both small intestine and colon tumors. Especially, ZD increased significantly the tumor incidence of colon in *Apc*^min/+^ mice. Our data, we believe, are the first time to indicate that ZD promotes carcinogenesis of colon tumors.

There are several conflicting studies on the effect of zinc deficiency on inflammation [26]. *In vitro* studies using different cell types and zinc concentrations and the effects of chelating agents have clearly shown that the impact of zinc cannot be explained unilaterally [27]. A study by Haase et al. has shown that zinc is necessary for activating the NF-κB signaling pathway induced by lipopolysaccharide (LPS), and that zinc interacts with the membrane-permeable zinc-specific chelator TPEN (N,N,N′,N′-tetrakis-(2-pyridyl-methyl) ethylenediamine) completely blocks this pathway [28]. On the other hand, a growing number of research articles support the role of zinc as a negative regulator of NF-κB signaling pathways, including our previous research [29, 30]. In addition, zinc supplementation significantly reduced the tumor burden of mice with multiple tumor suppressor deficiencies [31]. *In vitro* studies, zinc has an anticancer effect on non-small-cell lung cancer cells in the presence of functionally active p53 and enhances the efficacy of docetaxel in both p53-wild-type and p53-deficient cancer cells [32]. And the effect of zinc deficiency on lung cancer may be related to the activation of the Hedgehog (Hh) signaling pathway [33].

A variety of studies have shown that inflammatory bowel disease (IBD) such as ulcerative colitis and Crohn’s disease can promote the tumor malignant progression, invasion, and metastasis of CRC [34, 35]. CCL2, also known as MCP-1, mediates macrophage recruitment to promote tumor growth, progression, and metastasis. Retinoblastoma inactivation increases the secretion of the chemoattractant CCL2, which promotes tumor angiogenesis and recruitment of tumor-associated macrophages and myeloid-derived suppressor cells into the tumor microenvironment in several tumor types including sarcoma and breast cancer [36]. Furthermore, CCL2 affects the accumulation and function of myeloid-derived suppressor cells in the colon and tumor microenvironment during colon carcinogenesis [37]. CXC ligands (CXCLs) are composed of 14 members. In colon cancer, eight types of CXCL members have been identified including CXCL1, CXCL2, CXCL3, CXCL5, CXCL8, CXCL9, CXCL10, and CXCL12 [38]. According to the analysis of the human database, CXCL2 (called Gro-2/-β or MIP-2) increases in patients with colon cancer or ulcerative colitis [39]. It is shown that the expression of CXCL1, CXCL2, CXCL3, and CXCL8 is increased in CPT-11-R LoVo colon cancer cells. CXCL2 knocked down by short hairpin RNA results in reduced expression of cancer stem cells (CSCs) proteins, cyclins, epithelial to mesenchymal transition (EMT) markers, G proteins, and matrix metalloproteinases (MMPs) [5]. CXCL5, also known as epithelial neutrophil activating peptide 78 (ENA78), a member of the CXC-type chemokine family, is originally discovered as a potent chemoattractant and activator of neutrophil function [40]. Several lines of evidence indicate that CXCL5 participates in cancer-related inflammation, which triggers several aspects of malignancy [41]. Abnormal expression of CXCL5 has been found in many types of tumors. CXCL5 expression is increased in the highly metastatic hepatocellular carcinoma cell (HCC) lines and tumor tissues of patients. CXCL5 promotes proliferation, migration and invasion of HCC cells through the activation of the PI3K-Akt and ERK1/2 signaling pathways [42]. Multiple logistic regression analysis indicated that CXCL5 over-expression was associated with late gastric cancer and high N stage [43]. High expression of CXCL5 is associated with reduced overall survival in intrahepatic cholangiocarcinoma and hepatocellular carcinoma [44]. Especially, overexpression of CXCL5 enhanced the migration and invasion of colorectal cancer cells by inducing the epithelial-mesenchymal transition (EMT) through the activation of the ERK/Elk-1/Snail pathway and the AKT/GSK3β/β-catenin pathway [45].

CXCR2, a G-protein-coupled cell surface chemokine receptor, plays a vital role in the recruitment and response of the immune system. It mediates the neutrophil migration to the site of inflammation [46]. Eight ligands, CXCL1, 2, 3, 4, 5, 6, 7, and 8 of CXCR2 have been identified. These ligands have been shown to participate in the carcinogenesis in several types of cancer through CXCR2 [5, 47]. The expression of CXCR2 is a promoter of local and distant metastasis of CRC and unfavorably is associated with CRC patients’ prognosis [48]. CXCR2 is important in the immunoregulation of pancreatic cancer and inhibition of CXCR2 can reduce metastasis and improve response to gemcitabine and anti-PD1 [49]. Moreover, a single copy (heterozygote) deletion of the CXCR2 gene is sufficient to synergize with low-dose sulindac treatment in suppressing *Apc* min-induced intestinal polyposis [50].

Numerous inflammatory mediators have been implicated in cancer metastasis such as interleukin-6 (IL-6), tumor necrosis factor-alpha (TNF-α), and interleukin-1 beta (IL-1). IL-6 is secreted by various cell types, such as fibroblasts, endothelial cells, macrophages, T cells and myocytes. It is increasingly recognized that it is a critical cytokine linking chronic inflammation to cancer development [51, 52]. IL-1, a pro-inflammatory cytokine, has been identified as a potential biomarker for predicting breast cancer [53]. It has also been shown that IL-1β/NF-kb signaling promotes colorectal cancer cell growth through miR-181a/PTEN axis [54]. The level of TNF-α in the colon is dramatically increased in the mice with AOM/DSS-induced colon cancer [55].

COX-2 is overexpressed in different types of cancers, including lung, prostate, breast, and colorectal cancer [56–58]. According to our previous data, ZD-promoted esophageal cancer is associated with COX-2 up-regulation [8]. Non-steroidal anti-inflammatory drugs (NSAIDs) have been widely used to prevent inflammation-associated cancers. Celecoxib, a type of NSAIDs, has been evaluated as a chemopreventive drug by specifically inhibiting cyclooxygenase-2 (COX-2). It has been identified that celecoxib could reduce polyp size by altering the intestinal microbiota and metabolome of *Apc*^min/+^ mice [59]. Apart from the above, celecoxib performs a chemopreventive effect in colitis-mediated colon carcinogenesis [60]. In the present study, we found that celecoxib attenuated ZD-promoted colon tumors in *Apc*^min/+^ mice by suppressing inflammatory factors. To the best of our knowledge, this is the first data to provide evidence that ZD promotes colon tumor process in *Apc*^min/+^ mice.

In conclusion, our results have indicated that Zinc deficiency accelerates colon tumorigenesis by activating pro-inflammatory mediators. Celecoxib attenuated ZD-induced colon tumorigenesis by inhibiting the expression of pro-inflammatory factors. Our novel findings reveal the role of ZD in colon carcinogenesis and provide a potential preventive strategy.

## MATERIALS AND METHODS

### Animal and diets

Female weaning *Apc*^min/+^ mice (C57BL/6J background) were obtained from the animal Model Institution of Nanjing University, P.R. China. The mice were fed a standard laboratory diet under controlled temperature and a 12-hour light/dark cycle at 20-22° C. All animal procedures were approved by the Animal Care Committee of the Medical College of Nanchang University. Custom-formulated egg white-based diets were obtained from Harlan Teklad (Madison, WI, USA). The composition of zinc deficiency (ZD) (TD.85419) and zinc sufficiency diet (ZS) (TD.85420) were identical except for the Zn content, which was approximately 0.5 - 1.5 ppm and approximately 50.5 - 51.5 ppm, respectively. In all the groups, mice were fed ZD or ZS diet from 28 days old. Celecoxib (Pfizer, CA, USA), 6 mg/kg orally daily [60], was administrated on the same day of feeding the ZD/ZS diet.

### Experimental protocols

In the first experiment, to evaluate the role of ZD in mice survival, *Apc*^min/+^ mice were randomly divided into 2 groups, each with 20 mice: ZD and ZS diet groups. ZD or ZS diet was fed for 250 days (long term). The dead mice were counted daily.

In the second experiment, to test the effect of ZD on colon tumorigenesis, *Apc*^min/+^ mice were randomly divided into 2 groups, each with 15 mice: ZD and ZS diet groups. ZD or ZS diet was fed for 60 days (short term). The body weight was recorded every 15 days since the mice were fed ZD or ZS diet. At the end of the experiment, all mice were euthanized. Spleen, colon, small intestine and serum were separated. Spleen index was calculated.

In the third experiment, to evaluate the potential chemopreventive role of celecoxib in ZD-promoted intestinal cancer, *Apc*^min/+^ mice fed with ZD diet were treated with celecoxib, 6 mg/kg orally daily.

### Histopathological examination

Entire colons and small intestines were excised, cut longitudinally, and rinsed 3 times with ice-cold PBS. The small intestine was divided into three segments (proximal, middle and distal) equally according to the total length. Tumor numbers were counted and recorded under the dissecting microscope. The tumors were classified based on the diameter of the tumor.

### Real-time PCR quantification

Total RNA was isolated from the colon and small intestine with TRIzol (Invitrogen, Carlsbad, CA, USA) according to the manufacturer’s guidance. 1000 ng total RNA was used as a reverse transcription template using the Applied Biosystems Reverse Transcription Kit (Applied Biosystems, Foster City, CA, USA). Real-time quantitative PCR was performed in the ABI7500 real-time PCR system (Applied Biosystems, Foster City, CA, USA). The expression levels were normalized to psmb6.

### Enzyme-linked immunosorbent assay (ELISA)

CCL2, CXCL2, CXCL5, IL1β, IL6 and TNF-α were measured by ELISA kits (Abcam, Cambridge, MA, USA) as described by the manufacturer’s instruction in serum. The samples were analyzed in duplicate.

### Immunohistochemistry

Colons of 6 mice from each group were used. The paraffin-embedded tissues were cut into a thickness of 5 μm. Sections were used for immunohistochemistry (IHC) staining. The procedure is as follows: the paraffin sections were dewaxed and rehydrated. Antigen retrieval was performed by microwaving the sections in 0.01M sodium citrate buffer (pH 6.0). The endogenous peroxidase activity was blocked by 3% hydrogen peroxide (H_2_O_2_) for 30 min at room temperature and nonspecific binding sites were blocked. The slices were then incubated with primary antibodies against COX-2 (1:200; ab23672, Abcam), cyclin D1 (1:200; ab16663, Abcam), and PCNA (1:200; ab29, Abcam) overnight at 4° C. After washing three times in PBS, the sections were incubated with the HRP-conjugated Goat Anti-Rabbit IgG (PV-6001, ZSGB-BIO, China) for 1.5 h at room temperature. Immunoactivity was detected after incubation with diaminobenzidine and H_2_O_2_ for 2 min. Finally, sections were dehydrated in graded alcohols and mounted with neutral gums. Image-Pro Plus 6.0 image analysis software was used to analyze the average optical density of COX-2, cyclin D1, and PCNA. The immunostaining was blindly assessed.

### Western blot analysis

The tissues were lysed with RIPA buffer containing protease inhibitor cocktail. Total protein (50 μg) was loaded onto an SDS–PAGE and transferred to a polyvinylidene difluoride membrane. After incubating with anti-CXCR2 (1:1000; ab217314, Abcam, USA) and anti-COX-2 (1:1000; ab23672, Abcam, USA) antibodies overnight in a 4° C refrigerator, the membrane was transferred to the secondary antibody (1:5000; Cell Signaling Technology) incubating for 1 hour. Finally, an enhanced chemiluminescence reagent (Wanleibio, Shanghai, China) was used to detect the proteins on the membrane. Immunoreactivity was visualized using gel imaging system (BIO-RAD GelDoc XR+, USA). Image J software (National Institutes of Health, Bethesda, MD, USA) was used to analyze immunoblotting images. The relative protein levels were normalized to GAPDH (1:10000; Abcam).

### Statistical analysis

Data were presented as mean ± S.D. or mean ± S.E.M. Comparisons of mean values between two groups were carried out by using a t-test and those between multiple groups were subjected to one-way analysis of variance (ANOVA). Statistical significance was considered with P value less than 0.05.

## Supplementary Material

Supplementary Figure 1
