# Supplementary material for: Celecoxib alleviates zinc deficiency-promoted colon tumorigenesis through suppressing inflammation
Source: Aging (Albany NY). 2021 Mar 3;13(6):8320–34. doi: 10.18632/aging.202642 (PMC8034938; doi:10.18632/aging.202642)
Supplement: Supplementary Figure 1 [file aging-13-202642-s001.pdf]

## SUPPLEMENTARY FIGURE

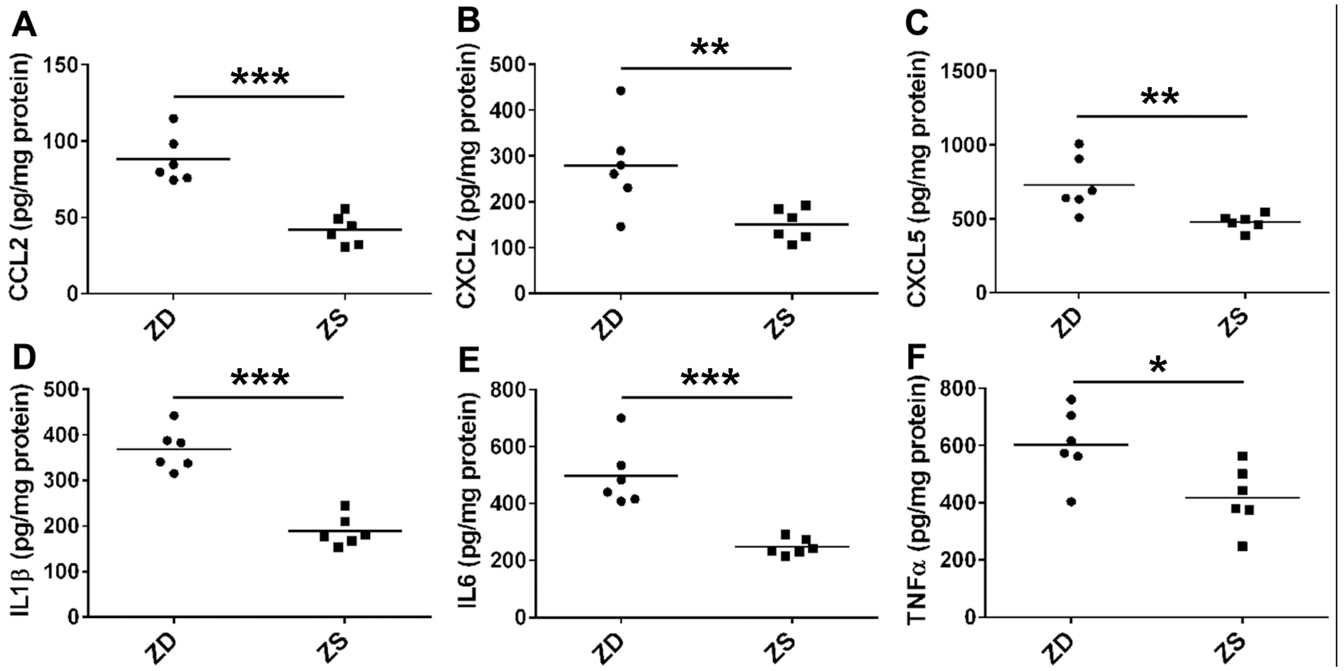


**Supplementary Figure 1. ZD induced the protein pro-inflammatory mediators in the distal small intestine**. (**A**) CCL2, (**B**) CXCL2, (**C**) CXCL5, (**D**) IL-1β, (**E**) IL6, and (**F**) TNF-α protein were measured by ELISA. Horizontal bars indicated the average protein levels. *$P < 0.05$, **$P < 0.01$, and ***$P < 0.001$ vs ZS group, n=6.
